# A Brief Journal of the Ordinary Progress of Natural Small-Pox, Both Distinct and Confluent

**Published:** 1816-06

**Authors:** Richard Walker

**Affiliations:** Oxford.


*For the London Medical and Physical Journal.*

### *A brief Journal of the ordinary Progress of Natural Small-pox, both Distinct and Confluent;* by RICHARD WALKER, M.D. of Oxford.

THE following journal is collected and arranged from cases of small-pox which have occurred under my own experience, and I flatter myself that the novelty and convenience of this form of arrangement, as an useful kind of *vade-mecum*, for the purpose of apprising or reminding a practitioner of the different circumstances in small-pox, in the progressive order as they occur, together with some observations of my own, which I consider as new, in the course of it, may not prove unacceptable.

Notwithstanding the zeal with which vaccination may be persevered in, I think I am warranted in predicting, from my late experience, that, however desirable the extinction or extermination of small-pox may be, this will never be completely effected; and that every place may still be liable to be visited by it at times; and, consequently, that any attempt at improving or facilitating the practice in it, may not be superfluous.

The small-pox is ordinarily divided into two kinds, viz. *distinct* and *confluent;* but, in reality, the difference consists only in a lesser or greater degree of virulence, or rather quantity or accumulation of pustules in each, constituting a greater degree of mild and safe sort, or a greater degree of virulent and dangerous sort;—the disease itself shewing, in different persons, every gradation, from the mildest or most distinct sort to the most virulent, or most confluent and dangerous sort; the gradations, in different persons, running insensibly one into the other: indeed it sometimes happens that pustules of the truly distinct kind, and those of the truly confluent kind, are to be seen in the same person. Moreover it is found that in inoculation, and in the casual or natural mode of taking small-pox, either may produce the other; from which it might be inferred, that the fluid or matter of small-pox itself is, in all instances, essentially the same in its nature; and that the greater or lesser degree of virulence or mildness depends entirely upon other circumstances, stances, viz. the natural constitution of the person, and the state of the habit at the time of taking it; the season of the year, the mode of treatment, &c.; and especially in the manner of receiving it, the method by inoculation unquestionably possessing a specific effect, independently of other circumstances, in producing a milder state of the disease than when it is taken naturally. The terms *vesicle* and *pustule* are used here almost indiscriminately: the former, however, applies to the eruption in its serous, the latter in its purulent, state.

1st.—The variolous or eruptive fever commences with an attack similar, in certain respects, to the attack of other fevers, viz. with rigors, succeeded by heat, thirst, pain in the head, back, and limbs, &c. The symptoms more especially belonging to this fever, and which indicate approaching small-pox, are languor, drowsiness, giddiness, sickness, and particularly a pain at the pit of the stomach, increased when pressed on, and vomiting; and, in children, starting in sleep, grinding of the teeth, and epileptic fits. The degree of mildness or violence in the ensuing small-pox may be commonly foreseen by an experienced practitioner, from the milder or more violent state of the symptoms enumerated. The more nearly the eruptive fever approaches to synocha, the milder or safer is the disease; and, the nearer it approximates to typhus, the greater will be the danger.

2d.—On the fourth day, and sometimes earlier, viz. on the third day, and, in some instances, on the second day, and even within twenty-four hours, from the first attack of the eruptive fever, the small-pox appears in the form of minute inflammatory spots, resembling flea-bites, first on the face, and successively on the neck and breast, the arms and body, and, lastly, the lower limbs. The later the appearance of the eruption, after the commencement of the febrile symptoms, ordinarily, the milder is the ensuing disease: thus, if the eruption does not appear until the fourth day (reckoning 24 hours for each day) from the first attack, which ordinarily happens about mid-day, but which is oftentimes not noticed till the evening, a mild or distinct kind of small-pox may be expected, and even if it happen on the third day, provided the febrile symptoms, &c. be not violent;* but, if the eruption

* It is the rule with the faculty to reckon the progress of the disease from the first attack; but it is sometimes desirable to refer to the date or age of the eruption: this may be done sufficiently near the truth, as a general rule, by abstracting two days from the former, by considering the third day of the disease the first of the eruption. Whatever mode of reckoning is used, the first day is always reckoned inclusively.

appear appear on the second day, a more virulent or confluent kind of small-pox may be expected, especially if the febrile attack be more violent, accompanied by a considerable prostration of strength, violent delirium or coma, indicating the fever to be of the typhus character; and, if the eruption appear on the first day, that is, in less than twenty-four hours, with an aggravation of the above symptoms, the worst degree of confluent small-pox may be expected, and very great danger apprehended.

3d.—By the fifth or sixth day, reckoning from the time of the first attack, which mode of reckoning is pursued throughout here, the eruption, in the distinct or mild sort, is ordinarily completed, and the patient becomes pretty well, or considerably better, although the giddiness sometimes continues, and is an indication that more are yet to come out. In the more violent state of the disease, when of the distinct kind, the patient still continues feverish and ill, and the more so in proportion as the small-pox is about to be of the more virulent or confluent kind; and the later is the period at which the eruption is completed.

4th.—By the sixth or seventh day, the pimples, if distinct, are somewhat elevated, inflamed and extended at their base, and have a minute vesicle, containing a colourless serous fluid at their apex, which is somewhat depressed. In the confluent kind, where they are so crowded in certain places as to run together, more or less in patches, which is mostly so in the face, they are flatter, and the small vesicle before-mentioned appears rather earlier than in the distinct sort; and, in the more confluent kind, the patches of pustules appear, at this time, like a skin, of a bright red or scarlet colour, applied, as it were, over the natural skin, or like one or more blisters.

5th.—By the seventh or eighth day, the pustules, if distinct, are much extended in size, viz. in the circumference of their base, with the inflammation around, and likewise in elevation. The face or head begins swelling, from the aggregate effect of the increasing inflammation, and enlargement of the pustules. This inflammation is in proportion to the quantity of pustules in the face and head, being scarcely apparent in those who have very few, and greater in proportion as the face or head is loaded with them, and sometimes sufficient, in the progress of the disease, by the tumefaction of the eye-lids, to close one or both eyes. A spitting, at this time, with some degree of hoarseness and cough, commences, in proportion to the quantity of pustules in the face, head, and throat, occasioning soreness in the throat, and difficulty in swallowing.

In the confluent kind, every one of these circumstances appear earlier, and are more aggravated, according to the greater degree of confluence which is most apparent in the face. From the state of the face, respecting the lesser or greater number of pustules, and their greater or lesser degree of confluence, the greater safety or danger of the patient is chiefly or almost exclusively prognosticated. Thus, in the confluent small-pox, the spitting before-mentioned amounts, in the progress of the disease, to an actual or copious salivation.—In children under the confluent kind of small-pox, other circumstances being the same, a diarrhœa ordinarily occurs, but not constantly, in place of the ptyalism or salivation which is incident to persons of an adult age,—although it sometimes happens that children have, like adults, the same propensity to salivation.

6th.—By the ninth or tenth day, in the distinct kind, the pustules in the face have arrived at maturity, viz. of their full hemispherical size, and contain purulent matter; the tumefaction of the face and head are at their height, and the patient, if at all loaded with pustules there, blind, by the bladder-like swelling of the eye-lids;—the spitting is at its height.

At this time, or rather before, the pustules in the upper limbs, (for the progress of the eruption is uniformly from upwards downwards,) viz. the arms and hands, approaching to maturity, occasion those parts to begin swelling, in like manner as the face had done a day or two before. About this time, the fever of maturation, or secondary fever, as it is called, comes on, which is scarcely perceptible in cases where there are few pustules; but evidently so in the fuller or more violent degrees of distinct small-pox, and sometimes even alarming.

In the confluent sort, the progress of the pustules to maturity is slower, and, indeed, in some instances, they never reach it; and the secondary fever, likewise, is more violent, approximating less or more to the typhoid character; and every circumstance enumerated in the distinct small-pox occurs here in a greater degree.

7th.—By the eleventh or twelfth day, in the distinct kind, the pustules, if of the mildest kind, are beginning to turn of a yellowish brown, and dry, in the face; or when less mild, burst, and discharge a yellowish matter, which concretes into a crust or scab, the pustule itself shrinking. The tumefaction of the face begins to subside, and the secondary fever abates. At this time the swelling of the arms and hands is at its height. But in the confluent kind, at the same period, the pustules, or rather patches of pustules, in the face, either remain remain crude and pallid, not unlike pieces of parchment laid on the natural skin, being scarcely elevated: or sooner or later they turn of a blackish brown, and which becomes progressively darker, and even blackish, in the worst degrees of confluent small-pox;*—the salivation, which was before thin and copious, is becoming thicker or more viscid, with increased cough and hoarseness.

8th.—By the thirteenth or fourteenth day, in the distinct kind and pretty full, the spitting has ceased, the swelling of the arms and hands have subsided, and the swelling of the legs and feet has commenced. In the confluent, the progress is the same, excepting that every circumstance is more violent in degree, the spitting more viscid, and the expectoration more difficult.

9th.—By the fourteenth or fifteenth day, in the distinct kind, if not much loaded, the pustules are become quite dry, and beginning to separate in the face, the tumefaction entirely subsided, and the patient perfectly recovered; the pustules in the body and limbs proceed in like manner quickly afterwards; the progress is progressively downwards, the pustules appearing and separating last on the feet, and towards the extremities, viz. where the circulation is most languid. In the confluent kind, at this time, and sometimes a day or two before, the critical time occurs, respecting recovery, the secondary fever, and all the dangerous circumstances, are at their height.

---

* About this time, and sometimes before, the state of the pustules in the cheeks and lower part of the face, in the more confluent kind of small-pox, are commonly rendered obscure, by the concretion of the serum which flows over those parts from the eyes and nose, giving the pustules there the appearance of having turned, whilst those in other parts of the face remain crude and pallid. About this time, and sometimes earlier, the petechiæ, or purple spots, appear in the interstices of the pustules, which, in the worst or most putrid kind of small-pox, degenerate into vesications, which burst, and exhibit a sphacelated appearance underneath, indicating almost certain death. Hæmorrhage, likewise, sometimes occurs, as bloody urine, &c. and, in some instances, even the fluid in the pustules is tinged with blood,—all signs of a great degree of putrescency. Even in the milder kind of confluent small-pox, the fluid in the pustules never acquires the perfectly purulent state which it does in the truly distinct kind; and yet this fluid, used for inoculation, may, and commonly does, under proper management, produce the mildest degree of distinct small-pox.

 10th.

10th.—After the fifteenth day, in the distinct kind, nothing more is to be looked for excepting a separation of the crusts or scabs, in progressive succession from the head downwards. In the confluent kind, if the patient survive the fifteenth day, (though they sometimes die earlier,) and the secondary fever and other threatening circumstances abate, especially if the swelling of the face and the upper and lower extremities has proceeded regularly, as the pustules in those parts have successively reached their height or maturity, great hopes may be entertained of the patient's recovery; but, if otherwise, the patient may die at some later period of the disease. The pustules, in the confluent kind, are much later in coming to maturity and separating than in the distinct kind, remaining sometimes on the feet, where they are ordinarily latest to be seen, until the twenty-second day, or afterwards; moreover, the pustules not unfrequently, in the more virulent kind of confluent small-pox, leave sores, extremely annoying, and long in healing;—such is rarely the case in the distinct or milder sort.

11th.—With respect to the *prognosis* in this disease, it may be collected from the different parts of the journal itself: thus, the variolous eruption will be less or more in quantity, or virulence, according to the lesser or greater degree of violence in the various circumstances accompanying the variolous or eruptive fever. Very severe pain in the head, back, and limbs, with much vomiting, are particularly indicative of a violent disease; and the ultimate event of the disease will be according to the quantity and nature of the pustules, a favourable termination being to be expected, with considerable confidence, in every instance of the truly distinct kind of small-pox, however full the patient may be with them. In the confluent kind, a greater degree of danger is to be apprehended; but where the confluence is inconsiderable, the event may be expected to be favourable. When they run very much together in the face, in large patches, the danger is greater, and when the face is, as it were, covered, or nearly so, with one patch, the danger is very great, as before-mentioned. The event, likewise, will depend upon, or be influenced by, various other circumstances, viz. the state of the habit of body; the age of the patient, very young infants, and particularly very old persons, having less vigour, are less able to resist or sustain a severe disease of long duration. The season of the year, likewise, influences the event very much, hot weather being extremely unfavourable; and, lastly, the mode of treatment, or rather management, of the patient, throughout its progress, particularly during the eruptive fever; and when the disease is violent, during the secondary fever, or fever of maturation.

With respect to the treatment of small-pox, I think it admits, for the convenience of practice, of being divided into three stages, viz. 1st, the eruptive fever, or indisposition previous to the eruption; 2dly, the eruption itself, and the progress of the eruption to maturity; and, 3dly, the fever of maturation, or secondary fever, which occurs at the crisis, or *turn*, as it is called, of the disease. On the first appearance, and throughout the progress of the variolous indisposition, when ascertained, or apprehended to be so, the principal intention should be to prevent, as far as possible, a great assimilation of variolous matter in the habit, or, in other words, to secure a mild or moderate eruption. This can ordinarily be effected, if the professional person be applied to in time, by a strictly antiphlogistic treatment, viz. a forbearance from every thing which excites inflammation, or heats the system; and the administration of cooling or mercurial purgatives; but, above all, by subjecting the patient, throughout this stage of the disease, as far as possible, to a uniformly cool temperature, and the use of cooling weak liquids.* In the outset of this, as in most other fevers, an antimonial emetic is ordinarily exhibited with advantage. With respect to blood-letting, great caution is requisite, lest the system should be so weakened thereby as to lay the foundation for a debilitated state, at the crisis, or towards the termination of the disease, when a vigorous state of the system may be required to enable the patient to pass through it. Moreover, if, from the nature of the symptoms, there is reason to apprehend the affection will be of the confluent kind, with a tendency, in the course of its progress, to typhus, blood-letting would be highly improper. It should be recollected, however, that excess or violence of *synocha* may, in the end, become *typhus:* hence, in some cases, a cautious blood-letting may be the means of averting typhus. On the proper treatment in this stage of the disease, particularly the cooling regimen in every respect, depends the favourable issue. Exposure to the cool air, without doors, possesses a specific effect, in itself, in this stage of the dis-

---

* During this period, and, indeed, throughout the disease, when they can be taken, acescent fruits, as oranges, cherries, &c. are both grateful to the palate, and salutary in effect.

ease, ease, in insuring a mild affection. The temperature should, as nearly as possible, be uniform: any transition from a cool temperature to a warm or hot one, is extremely improper; hence the patient, after having been subjected to cold air, as in winter, should not be permitted, as is too often the case, to approach the fire immediately, or soon after: for this produces a re-action of the system, a glow of heat, and, as I have frequently observed, influences the quantity and nature of the pustules very materially, a circumstance too commonly overlooked by the practitioner.

2dly, On the appearance of the eruptions, the same mode of treatment, particularly the cool temperature, and cooling regimen, is to be pursued, but, according to circumstances, with some degree of relaxation, until the eruption is completed. After this time, if the small-pox be of the distinct kind, and but moderately full, little more attention is required, and the patient may return nearly to an ordinary mode of living. But, if the patient be loaded with pustules, and the feverish indisposition remain, though abated, the cooling antiphlogistic plan must be adhered to, though less rigidly. In all instances of small-pox, during the time the pustules are approaching to maturation, unless feverish heat or any other circumstance forbid it, the patient should be supported, in order to nourish or fill out the pock, by a more liberal regimen than before, viz. a little animal food, and a little good beer, and cool air less rigidly enjoined; but every thing is still to be avoided which may tend to heat or inflame the patient,—hence the regimen should be of a light innocent nature. During this period it is essential, especially where there is a propensity to ptyalism, that the patient drink less or more plentifully of some demulcent nutritious liquids, somewhat warm, or not quite cold, as milk and water, barley water, &c. Costiveness is to be obviated, profuse diarrhœa checked, and an opiate administered over night, as occasion may require. The same mode of treatment may be adopted in the confluent kind, observing, however, that, when it is more virulent, and there is a more considerable degree of fever, but still of the nature of synocha, that there must be a closer or nearer adherence to the antiphlogistic plan.

3dly, When the pustules are matured, and the *turn*, as it is called, is approaching, the same mode of treatment is to be persisted in, unless any untoward circumstance happen, requiring a deviation from it: thus, if the secondary fever, as it is called, become urgent, as it occasionally does in the fuller kind of distinct small-pox, and more so in the more virulent virulent kind of confluent small-pox, but not of the typhus nature, the cooling antiphlogistic plan must be persisted in; but, if of the typhus nature, bark and mineral acids: still preserving a cooling temperature, supporting the patient, if necessary, with cordials, of which port-wine is the best.

The above mode of treatment, as I well know by ample and satisfactory experience, will not fail of conducting patients under small-pox safely, unless in cases of the most confluent and virulent kind; and in such cases I think there are but small hopes of success from the usual routine of practice commonly adopted, such as stuffing the patient with bark, and blistering him from head to foot, &c. I am well convinced that in this disease, as in some others, a due attention to regimen, and its appendages, conducted with judgment, is the more useful part of the medical art; and whoever neglects or misapplies this, deprives his patient of the benefit of the better part of his profession.—The nature of the variolous indisposition commonly denotes the character of the ensuing small-pox; but the eruption, when completed, more certainly developes it—then it may be perceived whether it will be of a mild, rank or virulent, or of a putrid tendency; and the plan of treatment must be adapted accordingly.

Should medicine be judged expedient or necessary in the progress of the disease, it will, of course, consist, under ordinary circumstances, in the administration of a neutral saline medicine, with the addition of a mild antimonial, during the feverish or inflammatory state of the patient, consistently with the antiphlogistic plan of regimen; and of a light preparation of the bark, during the progress of the filling and ripening of the pustules, consistently with the more liberal or invigorating regimen at that time.

In order for a practitioner to give his patient the best possible chance of recovery, in apparently critical or dangerous cases of small-pox, it is necessary he should have a prospective view, founded upon the present circumstances, viz. the age, constitution, and temperament of the patient, together with the nature of the affection, and other incidental contingencies, which may occur throughout the progress of the disease. By these he is prepared for the appearance of, and to obviate, any evil tendency or irregularity in the due progress of the disease, if possible, before the evil itself actually presents itself. A discerning practitioner, for instance, will, from present circumstances, foresee the probability of a retrocession of the small-pox, an irregularity respecting the swelling of the face or extremities, &c. and, likewise, be aware aware of the cause of it, and the most likely means of preventing it: this ordinarily arises from want of vigour in the habit, and is obviated by moderate warmth, and a restorative cordial regimen; and, if it actually occur, in addition to these, local stimulants, as fomentations, stimulating cataplasms, vesicatories, &c. The evils mentioned here, as likewise the confluent and malignant nature of the ensuing disease, not unfrequently arises from the mal-treatment of the relatives or attendants of the patient, respecting a heating stimulating regimen, in every respect, at the onset of the disease, before the practitioner is called in, the consequent evils of which it may be afterwards too late to obviate; but this must ever be attempted.

I shall conclude by allusion to a circumstance, which, if it were practicable, or should ever be found to be so, would, in my opinion, afford the greatest improvement in the treatment of small-pox, next to that of the cooling plan, adopted of late years, at the commencement of the disease. The danger and fatality in small-pox arises, ordinarily, from the secondary fever, which is produced by the absorption of the variolous matter, confined and stagnating in the pustules after maturation, aggravated or heightened by the loathsomeness and stench proceeding from those pustules, and, of course, contaminating the system. The only means, then, of effectually averting these evils, would be, as in the instances of other abscesses, of which the pustules collectively may be considered as an immense assemblage, so soon as they arrived at maturity, or rather before, to make an outlet for the constant discharge of the matter, keeping the part clean by sponging occasionally with warm water. It will be recollected that the pustules arrive at maturity not altogether, but progressively, which would diminish the difficulty or apparent impracticability of such treatment. Moreover, every prudent means should be used to keep the apartment as cool and pure as can be by ventilation, fumigation, &c.

With respect to physicking after small-pox, it should be considered that a more virulent or rank state of small-pox requires more cleansing after it than a milder sort; but, where symptoms of putrescency have occurred, leaving the patient in a debilitated state, bark and a restorative diet may be more required.

*April*, 1816.

*(To be concluded in our next.)*

